# Emotional Modulation of the Late Positive Potential during Picture Free Viewing in Older and Young Adults

**DOI:** 10.1371/journal.pone.0162323

**Published:** 2016-09-02

**Authors:** Jenna B. Renfroe, Margaret M. Bradley, Christopher T. Sege, Dawn Bowers

**Affiliations:** Department of Clinical and Health Psychology, University of Florida, Gainesville, Florida, United States of America; University of California, San Francisco, UNITED STATES

## Abstract

Hedonic bias during free viewing of novel emotional and neutral scenes was investigated in older adults and college students. A neurophysiological index of emotional picture processing–the amplitude of the centroparietal late positive potential (LPP)–was recorded from the scalp using a dense sensor array while participants (29 older adults; 21 college students) viewed emotionally engaging or mundane natural scenes that varied in specific content. Both students and older adults showed LPP enhancement when viewing affective, compared to neutral, scenes, and there was no difference in LPP amplitude between older individuals and college students when viewing neutral everyday scenes. However, compared to the college students, older individuals showed attenuated LPP amplitude when viewing emotional scenes, regardless of hedonic valence or specific content. Age related differences could be mediated by a reduction in reactive emotional arousal with age, possible mediated by repeated life exposure to emotional stimuli.

## Introduction

There is some evidence that older, relative to younger, adults show a positivity bias during emotional processing, as evidenced by reports of enhanced well being [1], better recall of positive autobiographical events [2], and longer duration of considering positive, relative to negative, attributes of a potential purchase [3]. In young adults, many studies have replicated the finding of an enhanced late positive potential (LPP) in the event-related potential measured over centroparietal sensors when viewing emotional (pleasant or unpleasant), compared to neutral, scenes, e.g.[4–13]. In the current study, we measured ERPs during free viewing of pictures that included a variety of emotional and neutral contents to assess hedonic bias in older adults and in college students.

A number of prior studies have measured event related potentials (ERPs) to assess whether older adults show electrophysiological evidence of preferential processing of pleasant stimuli, using a variety of different tasks and stimuli [14–18]. Evidence for a positivity bias in the LPP for older participants has been inferred from a number of diffferent patterns of modulation. For instance, Wood & Kisley (2006) [14] repeatedly presented two pictures of food (pleasant) and two pictures of mutilated bodies (unpleasant) in the context of an oddball categorization task in which participants decided whether the picture was pleasant, unpleasant, or neutral. Whereas younger adults demonstrated a larger LPP amplitude during unpleasant, compared to pleasant, picture viewing, older adults did not show this difference–this was interpreted as evidence for a positivity bias in older individuals. Using a similar paradigm, followup studies concluded that the difference was primarily mediated by reduced processing of unpleasant stimuli in older participants [15,17], and that higher cognitive functioning was associated with more reactivity to unpleasant scenes [18]. On the other hand, using three pictures of each hedonic content and a framing task, older adults showed specifically enhanced LPP amplitude for pleasant, compared to unpleasant, scenes regardless of framing instuctions, whereas younger individuals were influenced by the task [16]. Although each of these findings have been interpreted as supporting a positivity bias in older individuals, the pattern of ERP modulation providing evidence for that interpretation, as well as the paradigm, has varied across studies.

Because LPP modulation by emotion is reliably affected by variables such as picture repetition [19,20], task [21,22], specific picture content [13,23] and other types of cognitive demands [22,24], it could be informative to re-assess hedonic bias in older and younger participants by measuring the LPP when novel pictures varying in specific hedonic content are presented during free viewing–an experimental context in which many studies have replicated the finding that, in young adults, both pleasant and unpleasant scenes reliably elicit a larger LPP compared to everyday scenes. Thus, in the current study, LPP amplitude was measured during free viewing of pleasant, neutral, and unpleasant scenes. The goal was to determine whether there is evidence of a hedonic bias in older adults which, in previous studies, has been inferred from finding either an enhanced LPP when viewing pleasant, compared to unpleasant, scenes for older adults [14] or an enhanced LPP for unpleasant, compared to pleasant, scenes for younger, but not older adults. For young adults, we expected to replicate the established finding of enhanced LPP when viewing either pleasant or unpleasant, compared to neutral, scenes.

Previous studies measuring the late positive potential during free viewing in young adults have confirmed that highly arousing contents of erotica, romance, and human violence prompt the greatest enhancement of the LPP [13,23]. The 108 scenes presented in the current study were selected to represent 18 specific contents with 6 exemplars each, and so we next assessed the late positive potential as it varied with specific content to assess the extent to which any differences in the LPP might be mediated by this factor. Thus, the amplitude of the LPP was assessed as older individuals and college students viewed novel pictures depicting erotic couples, romance, nude women, nude men, recreation, snacks, loss, contamination, animal threat, animal threat, and mutilated bodies, together with neutral, scenes depicting everyday people, events, and objects.

## Materials and Methods

### Participants

Participants were 29 older adults (mean age 72 years) and 21 college students (mean age 20 years). The students were 18–22 year-old members of a General Psychology course at the University of Florida, recruited through the university’s online research participation system, who received class credit or financial compensation for participation. Older participants were respondents to flyers and mailings in nearby retirement communities, and received financial compensation. To be included in the older adult group, participants scored above a 25 on the Mini Mental State Examination. Exclusion criteria for both groups included any self-reported history of neurological or mental illness. Use of psychotropic medications was assessed for all participants, and no participants reported active use.

Questionnaire measures included various self-report measures of depression, apathy, and anxiety, including the Beck Depression Inventory-II (BDI-II) [25], the Apathy Scale [26], and the State/Trait Anxiety Inventory (STAI) [27].

### Materials and Design

Stimuli were 108 scenes selected from the International Affective Picture System [28]. Based on normative IAPS ratings, there were: 36 pleasant scenes (mean pleasure and arousal of 6.6 and 5.8 respectively); 36 neutral scenes (mean pleasure and arousal of 5.1 and 3.7 respectively), and; 36 unpleasant scenes (mean pleasure and arousal of 2.3 and 6.0 respectively). Pleasant and unpleasant pictures did not differ in normative arousal ratings. To explore the effect of picture content on LPP modulation, pictures were also selected in terms of the specific content depicted in each picture. Each hedonic category was thus subdivided (based on experimenter’s judgment) into 6 sets of 6 pictures, including: 1) erotic couples (IAPS nos. 4604, 4651, 4652, 4659, 4664, 4670); 2) romance (IAPS nos. 4599, 4606, 4609, 4623, 4641, 4660); 3) nude women (IAPS nos. 4180, 4232, 4250, 4255, 4290, 4310); 4) nude men (IAPS nos. 4470, 4490, 4520, 4530, 4531, 4550); 5) recreation (IAPS nos. 8158, 8163, 8191, 8193, 8300, 8341); 6) snacks (IAPS nos. 7220, 7230, 7270, 7350, 7405, 7560); 7) meals (IAPS nos. 7255, 7287, 7290, 7476, 7484, 7487); 8) animals (IAPS nos. 1121, 1313, 1350, 1670, 1908, 2309); 9) neutral faces (IAPS nos. 2190, 2200, 2210, 2214, 2230, 9070); 10) everyday events (IAPS nos. 2026, 2597, 7506, 7512, 7513, 8312); 11) neutral objects (IAPS nos. 7002, 7009, 7020, 7059, 7233, 7235); 12) everyday scenes (IAPS nos. 5471, 7248, 7249, 7546, 8325, 9468); 13) loss (IAPS nos. 2700, 2800, 2900, 3300, 9002, 9421); 14) contamination (IAPS nos. 2730, 7360, 9031, 9295, 9300, 9832); 15) accidents (IAPS nos. 9623, 9903, 9905, 9909, 9925, 9930); 16) animal threat (IAPS nos. 1050, 1120, 1201, 1300, 1304, 1930); 17) human threat (IAPS nos. 6250, 6260, 6510, 6550, 6570, 9413); and 18) mutilated bodies (IAPS nos. 3000, 3053, 3071, 3102, 9405, 9412).

Pictures were 1024 x 768 digitized color photographs presented approximately 13x16 inches in size on the screen. Participants sat approximately 36 inches from the computer monitor and were able to wear corrective lenses whenever necessary. Participants were shown six practice/exemplar images (two from each hedonic category) to assure that they were able to view and tolerate the images without difficulty. Each picture was presented for 3 s; pictures were presented in a counterbalanced event-related design (with no more than two of the same hedonic category occurring consecutively) and were separated by intertrial intervals that averaged three seconds (2–5 seconds).

### Data Recording

Electroencephalogram (EEG) was recorded at 250 Hz using an Electrical Geodesics Incorporated (EGI; Riverfront Research Park, 1600 Millrace Drive, Suite 307, Eugene, OR 97403, USA) 129 sensor net with a vertex reference. EEG signal was filtered on-line (0.01–48 Hz). Offline, data were reduced using EMEGS software [29]. Eye movements were corrected using BioSig, an open source software library for biomedical signal processing [30]. The data were lowpass filtered offline at 30 Hz, corrected for artifacts, interpolated using spherical splines [31], and re-referenced to the average reference. ERP averages included a mean of 36 trials per condition, with more than 80% of trials included in the average for each condition (pleasant, neutral, and unpleasant picture viewing). The number of rejected trials did not vary by age (*F*(1,48) = .25, *p* = .62) or hedonic content (*F*(2,96) = .87, *p* = .42).

### Experimental Procedure

Experimental testing took place at the Center for the Study of Emotion and Attention (CSEA) at the University of Florida. On the testing day, written informed consent was obtained from participants. Participants were given self-report questionnaires and then the EEG net was applied, followed by free viewing of emotional and neutral pictures. Participants were instructed to *“Simply view the pictures that are presented on the screen; please do not look away or close your eyes*, *but rather continue to view the picture the entire time it is presented on the screen*.*”*

After the picture viewing task was complete, participants rated the pictures for subjective experience of pleasure and arousal. Ratings were collected for two exemplars from each of the 18 content sub-categories, such that the same 12 pictures from each hedonic category (unpleasant, neutral, and pleasant) were rated by all participants. Pictures were rated using the self-assessment manikin (SAM) [32], in which ratings of pleasure and arousal are made on a scale from 1–9. Following the experiment, participants were paid and debriefed.

#### Data analysis

**Fig 1:**
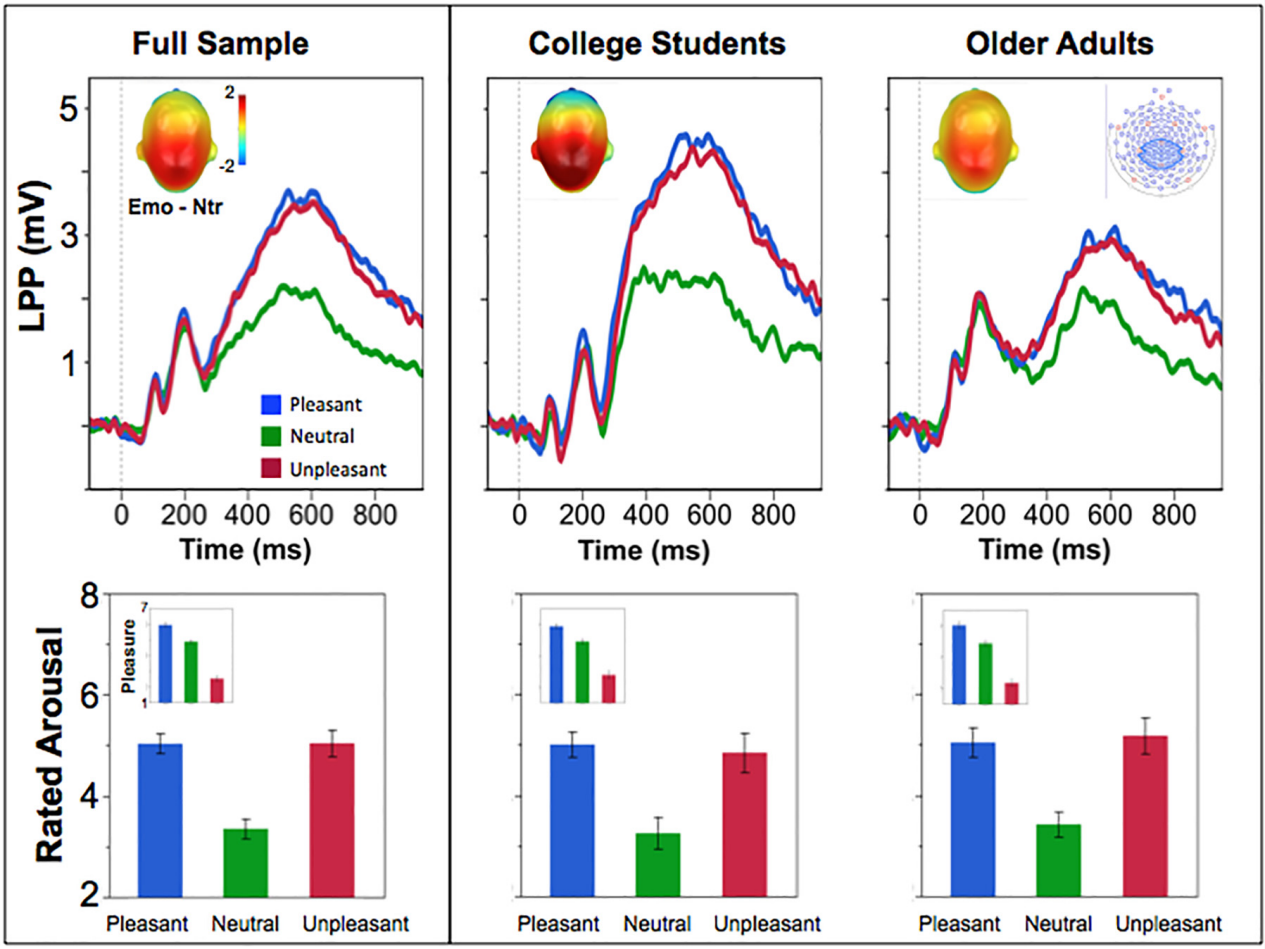
Event-related potential and ratings for pleasant, neutral, and unpleasant pictures. Top panel: Event-related potentials for a centro-parietal sensor group show the waveform when viewing pleasant, neutral or unpleasant scenes for the full sample, college students, and older participants. Insets show the centro-parietal location of the difference in the amplitude of the late positive potential (400–800 ms post picture-onset) when viewing emotional (pleasant or unpleasant), compared to neutral, scenes. Bottom panel: Mean ratings of emotional arousal (insets: pleasure ratings) for the full sample, college students, and older participants.

Sensors contributing to emotional modulation of the late positive potential were determined by averaging ERPs for emotional (pleasant and unpleasant) and neutral scenes across all participants, and plotting the difference in voltage in a 400–800 ms time window following picture onset on the scalp, as illustrated in Fig 1A (top, inset). For the full sample (and similarly for older individuals and for college students), affective modulation of the late positive potential was maximal over a group of centro-parietal sensors, consistent with many previous studies [4,13,21,23,33] (see Fig 1, upper insets). Accordingly, ERPs were averaged over a set of sensors that was consistent with previous research (31, 37, 42, 52, 53, 54, 55, 60, 61, 62, 67, 72, 77, 78, 79, 80, 85, 86, 87, 92, 93, 129), and were analyzed in subsequent mixed ANOVAs that assessed LPP amplitude in older participants and college students as it first varied with hedonic category (3: pleasant, neutral, unpleasant) and then with specific content (18: erotic couples, romance, nude women, nude men, recreation, snacks, meals, animals, neutral people, everyday scenes, neutral objects, everyday events, loss, contamination, accidents, human threat, animal threat, mutilated bodies).

## Results

### Demographics

Older adults were more educated than college students, whereas students endorsed slightly greater depression, apathy, and anxiety symptoms on self-report questionnaires, although symptoms were mild and not clinically significant for either group (Table 1). Additionally, these variables were not significant covariates and were thus not included in any subsequent analyses. Older adults scored 29 out of 30 on average on the MMSE, indicating that the older adult sample was not cognitively compromised.

**Table 1:** Mean (SD) demographic and questionnaire data for college students and older adults.

Measure	College Students	Older Adults	*p*-value
Age	19.7 (1.3)	72.5 (7.1)	*p* < .001
Education	13.1 (1.0)	16.5 (2.8)	*p* < .001
BDI-II	7.1 (6.2)	3.3 (3.5)	*p* < .05
Apathy Scale	11.8 (4.0)	9.1 (4.6)	*p* < .05
STAI state	29.0 (5.4)	27.3 (6.4)	*p* = .33
STAI trait	35.4 (9.0)	28.6 (6.5)	*p* < .01

### Hedonic Category: Pleasant, Neutral, Unpleasant

A 2 (Age: older, college) x 3 (Hedonic Category: unpleasant, neutral, pleasant) mixed-design ANOVA resulted in main effects of hedonic category for both ratings of pleasure, *F*(2,45) = 236.38, *p* < .001, *η*_*p*_^*2*^ = .83, and arousal, *F*(2,45) = 27.39, *p* < .001, *η*_*p*_^*2*^ = .37, but no effects involving age group. Unpleasant pictures were rated as less pleasant, *F*(1,47) = 186.17, *p* < .001, *η*_*p*_^*2*^ = .80, and more arousing, *F*(1,47) = 27.80, *p* < .001, *η*_*p*_^*2*^ = .37, than neutral scenes, and pleasant pictures were rated as more pleasant, *F*(1,47) = 70.47, *p* < .001, *η*_*p*_^*2*^ = .60, and arousing, *F*(1,47) = 81.58, *p* < .001, *η*_*p*_^*2*^ = .63, than neutral scenes (Fig 1, bottom panel).

A mixed-effects ANOVA with Age (2) and Hedonic Category (3) resulted in a significant Age x Category interaction, F(2,47) = 4.55, *p* = .02, *η*_*p*_^*2*^ = .09. LPP amplitude was significantly enhanced when viewing emotional compared to neutral scenes for both older adults, *F*(1,28) = 54.48, *p* < .001, *η*_*p*_^*2*^ = .66, and college students, *F*(1,20) = 29.96, *p* < .001, *η*_*p*_^*2*^ = .60 (Fig 1, top panel). Moreover, for both older adults and college students LPP enhancement compared to neutral scenes was found when viewing either pleasant (*F*(1,28) = 37.9, *p* < .001 and *F*(1,20) = 21.9, *p* < .001 for older adults and college students respectively) or unpleasant (*F(*1,28) = 37.4 *p* < .001 and *F*(1,20) = 31.8, *p* < .001 for older adults and college students respectively) pictures, and LPP during pleasant and unpleasant picture viewing did not differ for either age group. Rather, the significant interaction indicated that, whereas LPP amplitude was equivalent when older individuals and college students viewed neutral, everyday scenes, LPP amplitude was specifically attenuated when older participants, compared to college students, were viewing emotional scenes, *F*(1,48) = 7.88, *p* = .007, *η*_*p*_^*2*^ = .14. Thus, LPP amplitude was reduced for older adults, compared to college students, both when viewing pleasant scenes, *F*(1,48) = 6.62, *p* = .01, *η*_*p*_^*2*^ = .12, or unpleasant scenes, *F*(1,48) = 7.91, *p* = .007, *η*_*p*_^*2*^ = .14.

### Specific content

Table 2 lists the mean LPP amplitude as well as ratings of pleasure and arousal for older individuals and college students when viewing scenes depicting various specific contents. As expected, different specific contents differed in both rated pleasure, *F*(17,30) = 70.8, *p* < .001, and rated arousal, *F*(17,30) = 17.3, *p* < .001); however, consistent with the previous analysis, there were no effects involving Age for either the pleasure or the arousal ratings.

**Table 2:** Mean (SE) late positive potential amplitude, pleasure ratings, and arousal ratings for college students and older adults for specific contents.

	Late Positive Potential		Pleasure Ratings		Arousal Ratings	
Specific Content	College Students	Older Adult	College Students	Older Adult	College Students	Older Adult
Erotic Couples +	**5.5 (.6)**	**3.6 (.5)** *	6.8 (.3)	6.2 (.4)	6.4 (.4)	5.5 (.4)
Romance +	**5.2 (.6)**	**2.6 (.4)** *	6.0 (.3)	6.2 (.3)	4.6 (.3)	5.0 (.4)
Nude Women +	**6.5 (.7)**	**3.2 (.5)** *	7.0 (.3)	6.1 (.3)	6.5 (.3)	5.0 (.4)
Nude Men +	**4.2 (.5)**	**1.8 (.3)** *	3.0 (.4)	4.4 (.4)	3.0 (.4)	4.4 (.4)
Recreation +	**3.8 (.6)**	**1.6 (.4)** *	6.2 (.3)	6.7 (.3)	5.1 (.5)	5.5 (.4)
Animals ^	**3.4 (.6)**	**2.1 (.4)** *	5.3 (.3)	4.9 (.2)	3.2 (.4)	3.8 (.3)
Snacks +	1.7 (.6)	1.2 (.4)	6.3 (.3)	6.6 (.3)	4.4 (.4)	4.9 (.5)
Meals ^	2.6 (.5)	1.7 (.4)	5.2 (.2)	5.2 (.3)	3.4 (.3)	3.6 (.3)
Neutral Faces ^	2.1 (.7)	1.7 (.4)	4.6 (.3)	4.5 (.2)	3.4 (.4)	3.6 (.3)
Everyday Events ^	2.1 (.5)	1.7 (.4)	4.9 (.2)	5.1 (.2)	3.4 (.4)	3.5 (.3)
Everyday Objects ^	1.7 (.6)	1.0 (.3)	4.8 (.2)	4.8 (.2)	3.0 (.4)	2.9 (.3)
Everyday Scenes ^	0.4 (.5)	0.9 (.4)	4.7 (.2)	4.8 (.2)	3.2 (.5)	3.3 (.3)
Accidents -	1.6 (.6)	2.4 (.4)	3.2 (.3)	2.9 (.3)	5.1 (.5)	5.2 (.4)
Contamination -	3.0 (.5)	2.2 (.3)	3.0 (.4)	2.0 (.2)	3.6 (.4)	4.2 (.5)
Animal Threat -	4.4 (.7)	3.5 (.4)	3.5 (.4)	3.1 (.3)	5.0 (.4)	5.5 (.4)
Loss -	**2.8 (.6)**	**1.5 (.3)** *	2.8 (.3)	2.2 (.2)	3.8 (.4)	4.4 (.5)
Human Threat -	**4.8 (.7)**	**2.8 (.4)** *	2.8 (.3)	2.1 (.3)	6.1 (.5)	6.1 (.5)
Mutilation -	**4.7 (.6)**	**2.8 (.5)** *	1.5 (.2)	1.7 (.2)	5.5 (.6)	5.8 (.5)

Notes

+ Scenes included in pleasant average

^ Scenes included in neutral average

- Scenes included in unpleasant average

* Older adults show significant reduction in LPP amplitude, compared to college students.

For the amplitude of the LPP, a mixed-effects ANOVA using Age and Specific content (18) resulted in a significant Age x Content interaction, *F*(17, 29) = 4.00, *p* < .001. Followup comparisons indicated that, compared to college students, older individuals showed significant LPP reduction when viewing all of the pleasant contents, including romance, *F*(1,47) = 13.48, *p* < .001, *η*_*p*_^*2*^ = .22, erotic couples, *F*(1,47) = 6.50, *p* = .01, *η*_*p*_^*2*^ = .12, nude women, *F*(1,47) = 16.70, *p* < .001, *η*_*p*_^*2*^ = .27, nude men, *F*(1,47) = 19.34, *p* < .001, *η*_*p*_^*2*^ = .29, and recreation, *F*(1,47) = 10.54, *p* = .002, *η*_*p*_^*2*^ = .18. For unpleasant scenes, older adults, compared to college students, showed reduced LPP amplitude for scenes of mutilation, *F*(1,47) = 6.50, *p* = .01, *η*_*p*_^*2*^ = .12, human threat, *F*(1,47) = 6.74, *p* = .01, *η*_*p*_^*2*^ = .13, and loss, *F*(1,47) = 4.79, *p* = .03, *η*_*p*_^*2*^ = .09. For all of the neutral contents, there were no differences in LPP amplitude as a function of age.

## Discussion

There was no evidence of hedonic bias in LPP amplitude during free viewing of novel emotional scenes in older adults or in college students. Rather, for both groups of individuals, amplitude of the LPP was enhanced when viewing emotionally engaging, compared to neutral, scenes regardless of hedonic valence. Thus, LPP amplitude was significantly enhanced when viewing pleasant or unpleasant, compared to neutral, scenes, for both college students and older individuals, with neither group showing a significant difference in LPP enhancement when viewing pleasant or unpleasant scenes. Rather, older participants and college students differed primarily in that older adults showed attenuated LPP amplitude specifically when viewing emotional scenes, which was not accompanied by differences in evaluative ratings of pleasure or arousal.

Finding reduced LPP amplitude during unpleasant picture viewing for older individuals, compared to college students, is consistent with previous investigations reporting this LPP difference between older and younger participants in the context of an oddball categorization task [15,17]. In the current study, LPP amplitude was significantly attenuated for older adults when viewing highly arousing scenes of mutilation, which agrees well with Wood & Kisley (2006) [15], who found a similar between-group attenuation of the LPP when 2 scenes of mutilation comprised the unpleasant content. Moreover, a reduction in LPP amplitude for older adults, compared to college students, was also found for unpleasant scenes of human threat and loss, although scenes of accidents and contamination did not result in enhanced LPP, compared to neutral scenes, for either college students or older participants.

Replicating Rehmert and Kisley (2013) [16], older individuals showed enhanced LPP amplitude when viewing pleasant, compared to neutral, scenes in the current study. On the other hand, Rehmert and Kisley (2013) [16] also report a positivity bias specifically for older adults, evidenced by enhanced LPP amplitude for pleasant, compared to unpleasant, scenes, which was not found here. In that study, LPP amplitude was compared for 3 pleasant exemplars (children swimming, puppies, Disney castle) and 3 unpleasant exemplars (sad children, roach on pizza, car accident). Interestingly, scenes of accidents and contamination did not prompt differential LPP amplitude for older individuals or college students in the current study, which could contribute to the difference found in the earlier study. On the other hand, additional variations from the prototypical paradigm, including the number and content of scenes, repetition, oddball context, and task requirements are additional factors that could impact the observed pattern of LPP modulation.

The current data primarily indicate that, in a free viewing context that presents multiple novel exemplars representing a variety of emotional scenes of different content, differences in hedonic bias between older individuals and college students are not strongly apparent. And, whereas some studies have found evidence for a negativity bias in younger individuals, e.g., [15,34,35], the lack of hedonic bias found for college students in the current study replicates previous data that similarly report no evidence of bias when a variety of pleasant and unpleasant contents are included in the stimulus set [12]. Rather, the current data suggest that, regardless of age, both pleasant and unpleasant contents are emotionally engaging, as evidenced by equivalent amplitude of the LPP for pleasant and unpleasant contents that include a number of different specific contents.

Attenuated general emotional reactivity for older, compared to younger, adults is consistent with an interpretation of reduced physiological emotional arousal with aging, as suggested by previous reports in which, compared to young adults, older adults show reduced peripheral physiology (heart rate, finger temperature) when reliving past emotional experiences [36]. Additionally, Gavazzeni, Wiens, and Fischer (2008) [37] reported that older adults show reduced skin conductance responses when viewing unpleasant pictures compared to young adults (the study did not include pleasant pictures). Most relevant, Langeslag and van Strien (2009) [38] found reduced LPPs for older, compared to younger, individuals in an intentional memory task that involved emotional scenes. Moreover, whereas physiological arousal was reduced for older adults in these studies, subjective rated arousal was not, suggesting that physiology might be a more sensitive indicator of age-related habituation than self-report [36–38].

Reduction in physiological emotional arousal for older individuals (as well as, perhaps, hyper-engagement for younger participants) could be mediated by differential lifetime exposure to emotional scenes. Thus, aging almost certainly involves increased exposure to a variety of emotional scenes across the lifespan, and studies have found that affective modulation of the LPP is retained, but greatly attenuated, with repeated presentation of the same picture, either within or between sessions,e.g., [19,20]. If older adults, who have lived longer, show an attenuated LPP amplitude primarily because of lifetime exposure, a hypothesis of heightened arousal for novel stimuli in college students is also a reasonable interpretation. One way to assess this speculation in the future would be to measure LPP amplitude following picture repetition, as it predicts that differences between younger and older individuals will be larger for novel then for repeated scenes.

As found in previous studies, older adults in the current sample endorsed higher levels of positive affect [1] as evidenced by reporting significantly fewer symptoms of depression, anxiety, and amotivation, compared to the college students. Thus, attenuated LPP amplitude found for aversive scenes in the older individuals may promote a psychologically healthy advantage in which aging is associated with heightened sense of well being as suggested by socioemotional selectivity theory [39]. On the other hand, a similar reduction in LPP amplitude when viewing pleasant scenes is less easily explained by this account. There is some evidence, however, that older individuals specifically find pleasant scenes less arousing than younger individuals [32,40]. Thus, it is possible that pleasant scenes are less arousing for older individuals, and that a neurophysiological measure–amplitude of the late positive potential–is a more sensitive index of habituation than evaluative reports, which can be affected by demand, subjective interpretations, and other voluntary responses in the experimental setting, and thus often do not correspond to differences in physiological arousal across individuals,e.g., [41,42]. Furthermore, the results of the current study suggest that older adults show reduced physiological response across a broad range of pleasant stimuli, and that this effect is not due to differences in responses to one particualar appetetive category, such as erotica.

In summary, there was little evidence of a specific hedonic bias in older individuals when emotional engagement was measured as the amplitude of the cortically-measured LPP in a prototypical free viewing paradigm that presented a variety of novel pleasant and unpleasant contents. Rather, both older individuals and college students showed an enhanced LPP when viewing a variety of emotionally arousing scenes, relative to neutral, everyday scenes. Compared to college students, older adults showed attenuated late positive potentials specifically when viewing emotional scenes, and this attenuation did not differ depending upon whether hedonic valence was pleasant or unpleasant. A reduction in the LPP for older participants was found for scenes depicting a variety of different affective contents, which could be mediated by more frequent *in vivo* exposure across the lifespan for older individuals. Greater exposure to scenes depicting a variety of positive and negative life events could prompt relative habituation in LPP amplitude, as found, for instance, when college students view repeated picture presentations. A reduction in defensive engagement for unpleasant stimuli in particular could be advantageous, in that it could contribute to the self-reported higher levels of well being and positive affect in older adults.
